# Survey of zoonotic parasites and bacteria in faeces of Canada geese (*Branta canadensis*) in North‐Central Oklahoma

**DOI:** 10.1002/vms3.791

**Published:** 2022-03-22

**Authors:** Yoko Nagamori, Marisa A. Litherland, Nicole R. Koons, Anna R. Linthicum, Akhilesh Ramachandran

**Affiliations:** ^1^ Department of Veterinary Pathobiology, College of Veterinary Medicine Oklahoma State University Stillwater Oklahoma; ^2^ Oklahoma Animal Disease Diagnostic Laboratory, College of Veterinary Medicine Oklahoma State University Stillwater Oklahoma

**Keywords:** antimicrobial resistance, *Campylobacter*, *Cryptosporidium*, *Giardia*, *Salmonella*

## Abstract

**Background:**

As a population of non‐migratory Canada geese (*Branta canadensis*) has been growing in residential and recreational areas, public concerns on potential acquisition of zoonotic pathogens from Canada geese and their faecal deposits have been increasing.

**Objectives:**

The main study objective was to evaluate the prevalence of zoonotic microorganisms, *Campylobacter* spp.*, Cryptosporidium* spp.*, Giardia* spp. and *Salmonella* spp. and antimicrobial resistant *Escherichia coli* in faeces of Canada geese residing in North‐Central Oklahoma, United States.

**Methods:**

A total of 204 faecal samples were collected from 11 locations in North‐Central Oklahoma, where public recreational areas such as lakes and ponds were located, and Canada geese were commonly inhabited. Faecal samples were examined by a centrifugal flotation to evaluate the prevalence of *Cryptosporidium* spp. and *Giardia* spp. infections. A total of 180 faecal samples were grouped into 36 pooled samples and cultured using standard culture methods to detect the prevalence of *Campylobacter* spp. and *Salmonella* spp. infections. The antimicrobial resistance profile was determined on 32 *E. coli* isolates recovered from the 36 sample pools, using the Kirby Bauer Disk Diffusion method.

**Results:**

The targeted zoonotic pathogens were not identified by the faecal examinations performed. Of the 32 *E. coli* isolates, 17 isolates (53.1%) demonstrated resistance to ≥1 antimicrobial agent.

**Conclusions:**

Targeted zoonotic pathogens were not detected among the examined resident Canada geese in North‐Central Oklahoma. The findings of multiple‐antimicrobial resistant *E. coli* infections are potentially a public health concern although the prevalence was low in this study. Further, larger scale surveys are recommended.

## 1 INTRODUCTION

Populations of resident (non‐migratory) Canada geese (*Branta canadensis*) have been growing significantly among urban sites throughout North‐Central Oklahoma, as well as many other regions of North America, due to several factors including the lack of predators and the availability of suitable habitats for geese, such as artificial ponds and lakes surrounded by well‐maintained turfgrasses (Ayers et al., 2014; Cole et al., 2005; Gorham & Lee, 2016; Leonard, 2013; Rutledge et al., 2013; Vogt et al., 2018; Zhou et al., 2004). Environment Canada (2011) estimated that there are at least seven million Canada geese present in North America, and its population is stable or increasing. The faecal droppings of the waterfowl are commonly found in close proximity with populated public recreational areas, causing a source of environmental contamination and an increase of potential public health concerns for humans. A large number of microorganisms have been recovered from the faeces of Canada geese, including *Campylobacter* spp., *Cryptosporidium* spp., *Giardia* spp.*, Salmonella* spp. and antimicrobial resistant *Escherichia coli* (Ayers et al., 2014; Cole et al., 2005; Converse et al., 1999; Fallacara et al., 2001; Fallacara et al., 2004; Gorham & Lee, 2016; Graczyk et al., 1998; Kassa et al., 2001; Kassa et al., 2004; Moriarty et al., 2011; Rutledge et al., 2013; Vogt et al., 2018; Zhou et al., 2004). Humans can contract these microorganisms through accidental ingestion from contaminated environments during leisure activities such as swimming, playing in sediments, fishing, water skiing and picnicking near beaches where Canada geese are often observed (Acha & Szyfres, 2003; Ayers et al., 2014; Center for Disease Control and Prevention, 2019; Center for Disease Control and Prevention, 2020; Rutledge et al., 2013).

The previously reported prevalence of microorganisms in Canada geese was variable, ranging from 4.6% to 51.8% for *Campylobacter* spp. (Fallacara et al., 2001; Fallacara et al., 2004; Kassa et al., 2001; Moriarty et al., 2011; Rutledge et al., 2013; Vogt et al., 2018), 5.0–90.0% for *Cryptosporidium* spp. (Converse et al., 1999; Graczyk et al., 1998; Kassa et al., 2001; Kassa et al., 2004; Moriarty et al., 2011; Zhou et al., 2004), 0.0–100.0% for *Giardia* spp. (Ayers et al., 2014; Converse et al., 1999; Graczyk et al., 1998; Kassa et al., 2001), 0.0–2.3% for *Salmonella* spp. (Converse et al., 1999; Fallacara et al., 2004; Vogt et al., 2018) and 0.0–100.0% for antimicrobial resistant *E. coli* (Cole et al., 2005; Fallacara et al., 2001; Vogt et al., 2018). As discussed in previous studies (Benskin et al., 2009; Vogt et al., 2020), these variations can be greatly influenced by various factors, such as different sampling and testing methods and differences in sample collection by geographical regions, seasons, length of study (i.e. months vs. years), sources (i.e. live vs. dead birds) and effective sample sizes. Although the prevalence of these pathogens recovered from the faeces of Canada geese has been reported in several states of the United States, including Colorado, Illinois, Maryland, Massachusetts, New Jersey, North Carolina, Ohio and Virginia (Ayers et al., 2014; Converse et al., 1999; Fallacara et al., 2001; Fallacara et al., 2004; Gorham & Lee, 2016; Graczyk et al., 1998; Kassa et al., 2001; Kassa et al., 2004; Rutledge et al., 2013; Zhou et al., 2004), no survey has been conducted in the South‐Central region of the United States. Our main study objectives were to evaluate the prevalence of zoonotic pathogens, specifically focused on *Campylobacter* spp., *Cryptosporidium* spp., *Giardia* spp. and *Salmonella* spp., and antimicrobial resistant *E. coli* in the faeces of Canada geese inhabiting North‐Central Oklahoma and to assess the concerns for Canada geese as a potential public health threat. Additionally, the prevalence of other parasitic and bacterial infections in the resident Canada geese in the same area was determined.

## 2 MATERIALS AND METHODS

### 2.1 Sample collection

Between May and July 2019, faecal droppings were collected from Canada geese observed in 11 recreational places located in three counties within North‐Central Oklahoma (OK): five locations (L‐1 through L‐5) in Stillwater, Payne county (Table 1); four locations (L‐6 through L‐9) in Oklahoma City and Edmond, Oklahoma county (Table 2) and two locations (L‐10 and L‐11) in Broken Arrow, Tulsa county (Table 3). At each recreational area, geese were seen to associate freely with the human population.

**TABLE 1:** Prevalence of intestinal parasites identified in faecal samples of Canada geese inhabited in Stillwater, Payne county

	Sampling locations in Stillwater, Payne county (total number of samples)										Total (*n* = 96)	
	L‐1 (*n* = 28)		L‐2 (*n* = 6)		L‐3 (*n* = 23)		L‐4 (*n* = 18)		L‐5 (*n* = 21)			
Parasites	Positive number	Prevalence % (95% CI)	Positive number	Prevalence % (95% CI)	Positive number	Prevalence % (95% CI)	Positive number	Prevalence % (95% CI)	Positive number	Prevalence % (95% CI)	Positive number	Prevalence % (95% CI)
Ascarid eggs	8	28.6 (14.2–48.2)	1	1.7 (0.9–58.9)	10	43.5 (24.7–64.0)	6	33.3 (15.6–58.6)	13	61.9 (40.3–80.3)	38	39.6 (30.1–50.0)
*Capillaria* eggs	0	0.0 (0.0–11.9)	0	0.0 (0.0–41.1)	0	0.0 (0.0–14.5)	0	0.0 (0.0–18.5)	0	0.0 (0.0–15.9)	0	0.0 (0.0–3.91)
Cestode eggs	0	0.0 (0.0–11.9)	0	0.0 (0.0–41.1)	1	4.3 (0.2–21.3)	0	0.0 (0.0–18.5)	0	0.0 (0.0–15.9)	1	1.0 (0.06–5.6)
*Cryptosporidium* oocysts	0	0.0 (0.0–11.9)	0	0.0 (0.0–41.1)	0	0.0 (0.0–14.5)	0	0.0 (0.0–18.5)	0	0.0 (0.0–15.9)	0	0.0 (0.0–3.91)
*Giardia* cyst/trophozoites	0	0.0 (0.0–11.9)	0	0.0 (0.0–41.1)	0	0.0 (0.0–14.5)	0	0.0 (0.0–18.5)	0	0.0 (0.0–15.9)	0	0.0 (0.0–3.91)
Coccidian oocysts	16	57.1 (38.1–74.2)	6	100.0 (58.9–100.0)	18	78.2 (56.7–91.0)	8	44.4 (23.7–67.0)	21	100.0 (84.1–100.0)	69	71.9 (62.0–80.3)
Schistosome eggs	0	0.0 (0.0–11.9)	0	0.0 (0.0–41.1)	0	0.0 (0.0–14.5)	1	5.6 (0.3–27.1)	0	0.0 (0.0–15.9)	1	0.0 (0.0–3.91)
Strongylid eggs	6	21.4 (9.8–40.9)	1	1.7 (0.9–58.9)	3	13.0 (3.7–32.4)	2	11.1 (2.0–33.0)	7	33.3 (15.9–55.1)	19	19.8 (12.9–29.1)
*Strongyloides* eggs	0	0.0 (0.0–11.9)	0	0.0 (0.0–41.1)	1	4.3 (0.2–21.3)	0	0.0 (0.0–18.5)	0	0.0 (0.0–15.9)	1	0.0 (0.0–3.91)
Total parasites detected	30	n/a	8	n/a	33	n/a	17	n/a	41	n/a	129	n/a

*Note*: A total of 129 parasites with six different parasite groups/genera were detected in 96 faecal samples.

**TABLE 2:** Prevalence of intestinal parasites identified in faecal samples of Canada geese inhabited in Edmond, Norman and Oklahoma City, Oklahoma county

	Sampling locations in Edmond, Norman and Oklahoma City, Oklahoma county (total number of samples)								Total (*n* = 78)	
	L‐6 (*n* = 39)		L‐7 (*n* = 24)		L‐8 (*n* = 4)		L‐9 (*n* = 11)			
Parasites	Positive number	Prevalence % (95% CI)	Positive number	Prevalence % (95% CI)	Positive number	Prevalence % (95% CI)	Positive number	Prevalence % (95% CI)	Positive number	Prevalence % (95% CI)
Ascarid eggs	16	41.0 (26.7–57.8)	12	50.0 (31.0–69.0)	3	75.0 (24.9–98.7)	6	54.5 (26.5–80.0)	37	47.4 (36.5–58.8)
*Capillaria* eggs	0	0.0 (0.0–8.6)	0	0.0 (0.0–13.9)	0	0.0 (0.0–52.7)	3	27.3 (7.9–59.6)	3	3.8 (1.1–10.7)
Cestode eggs	1	2.6 (0.1–13.6)	0	0.0 (0.0–13.9)	0	0.0 (0.0–52.7)	0	0.0 (0.0–26.5)	1	1.3 (0.07–6.83)
*Cryptosporidium* oocysts	0	0.0 (0.0–8.6)	0	0.0 (0.0–13.9)	0	0.0 (0.0–52.7)	0	0.0 (0.0–26.5)	0	0.0 (0.0–4.8)
*Giardia* cyst/trophozoites	0	0.0 (0.0–11.9)	0	0.0 (0.0–13.9)	0	0.0 (0.0–52.7)	0	0.0 (0.0–26.5)	0	0.0 (0.0–4.8)
Coccidian oocysts	35^*^	89.7 (75.9–96.4)	12	50.0 (31.0–69.0)	4	100.0 (47.3–100.0)	7	63.6 (33.3–86.5)	58	73.1 (62.2–82.2)
Schistosome eggs	0	0.0 0.0–8.6)	0	0.0 (0.0–13.9)	0	0.0 (0.0–52.7)	0	0.0 (0.0–26.5)	0	0.0 (0.0–4.8)
Strongylid eggs	4	10.3 (3.6–24.1)	3	12.5 (3.5–31.0)	0	0.0 (0.0–52.7)	3	27.3 (7.9–59.6)	10	12.8 (6.8–22.3)
*Strongyloides* eggs	1	2.6 (0.1–13.6)	0	0.0 (0.0–13.9)	0	0.0 (0.0–52.7)	1	9.0 (0.5–40.4)	2	2.6 (0.5–8.8)
Total parasites detected	57	n/a	27	n/a	7	n/a	20	n/a	111	n/a

*Note*: A total of 111 parasites with seven different parasite groups/genera were detected in 78 faecal samples.

* Of 35 coccidia samples, one sample collected from the L‐7 contained *Isospora* spp. oocysts.

**TABLE 3:** Prevalence of intestinal parasites identified in faecal samples of Canada geese inhabited in Broken Arrow, Tulsa county

	Sampling locations in Broken Arrow, Tulsa county (total number of samples)				Total (*n* = 30)	
	L‐10 (*n* = 19)		L‐11 (*n* = 11)			
Parasites	Positive number	Prevalence % (95% CI)	Positive number	Prevalence % (95% CI)	Positive number	Prevalence % (95% CI)
Ascarid eggs	3	15.8 (4.4–39.2)	5	45.5 (20.0–73.5)	8	26.7 (13.1–44.9)
*Capillaria* eggs	0	0.0 (0.0–17.6)	0	0.0 (0.0–26.5)	0	0.0 (0.0–11.2)
Cestode eggs	0	0.0 (0.0–17.6)	0	0.0 (0.0–26.5)	0	0.0 (0.0–11.2)
*Cryptosporidium* oocysts	0	0.0 (0.0–17.6)	0	0.0 (0.0–26.5)	0	0.0 (0.0–11.2)
*Giardia* cyst/trophozoites	0	0.0 (0.0–17.6)	0	0.0 (0.0–26.5)	0	0.0 (0.0–11.2)
Coccidian oocysts	16	84.2 (60.8–95.6)	10	90.0 (59.6–99.5)	26	86.7 (70.2–95.3)
Schistosome eggs	0	0.0 (0.0–17.6)	0	0.0 (0.0–26.5)	0	0.0 (0.0–11.2)
Strongylid eggs	7	36.8 (17.6–60.8)	2	18.2 (3.3–50.0)	9	30.0 (16.3–48.3)
*Strongyloides* eggs	0	0.0 (0.0–17.6)	0	0.0 (0.0–26.5)	0	0.0 (0.0–11.2)
Total parasites detected	26	n/a	17	n/a	43	n/a

*Note*: A total of 43 parasites with three different parasite groups/genera were detected in 30 faecal samples.

At each sampling visit, Canada geese were directly monitored for the moment of defecation for up to 2 h. Immediately after defecation by a goose was observed, a whole portion of freshly voided faeces was collected. When faeces was watery, a plastic disposable spoon was utilised. Although multiple samplings from the same goose at visit were avoided, identification or tracking of individual geese was not conducted in this study. The number of faecal samples collected per visit varied from four to 24 samples due to variable number of geese present at each site during the sampling. Individual faecal samples were stored separately in a plastic bag, and all samples were kept at 4°C until examinations.

### 2.2 Parasitic examination

A total of 204 faecal samples were collected from Canada geese: 96 from Payne county, 78 from Oklahoma county and 30 from Tulsa county. Within 7 days of the collection date, a centrifugal faecal flotation test with Sheather's sugar solution (specific gravity of 1.25) was performed on each sample to detect the evidence of *Cryptosporidium* spp. and other parasitic infections (Zajac & Conboy, 2012). Approximately 1–4 g of faeces was mixed with about 15 ml of Sheather's sugar solution, and the mixture solution was then strained and placed into a 15‐ml conical centrifuge tube until a convex meniscus was formed. A coverslip was placed on the tube, and the mixture was centrifuged in a Centra CL2 centrifuge (Thermo Fisher Scientific; Waltham, Massachusetts, USA) at a speed of about 500–650 × *g* for 5 min. The coverslip was then removed and placed onto a glass slide and examined microscopically for parasites. The slide was scanned at a 100× magnification and periodically read at 200× and 400× magnifications for small protozoan parasites. Any parasite stages observed were morphologically identified, narrowed down to a taxonomic group of parasites or genus level when possible and recorded for data analysis (Soulsby, 1982; Zajac & Conboy, 2012). In addition to a centrifugal flotation with Sheather's sugar solution on each sample, three to seven faecal samples collected from the same location at the same date were pooled and examined by an additional centrifugal flotation with a 33% zinc sulphate solution (specific gravity of 1.18) to identify *Giardia* spp. trophozoites/cysts. All faecal slides were read by a board‐certified parasitologist and a veterinary student with assistance from the parasitologist.

### 2.3 Bacterial isolation

Several faecal samples were removed from bacterial evaluations due to improper sample handling; thus, a total of 180 faecal samples were grouped into 36 pools and cultured for bacterial growth. Pools were made up of four (11 pools), five (14 pools) or six (11 pools) individual faecal samples recovered from the same location on the same day. All cultures were performed within 24 h of sample collections.

Aerobic culture was performed using 5% sheep blood agar and MacConkey agar (Hardy Diagnostics; Santa Maria, California, USA) at 37°C in 5% CO_2_ environment. Cultures were monitored for bacterial growth for 2 days. A direct culture method was followed for *Campylobacter* spp. culture as this method has been evaluated and found to be useful for *Campylobacter* spp. enumeration (Oyarzabal et al., 2005). Each sample pool was streaked onto Campy FDA agar (Hardy Diagnostics; Santa Maria, California, USA) and incubated in a microaerophilic environment at 42°C for 7 days. Culture plates were observed for bacterial growth every other day following incubation. A selective enrichment culture was performed for *Salmonella* spp. detection. Approximately 10% (w/v) of pooled faecal sample was added into Tetrathionate Broth Base, Hajna (90 ml) (HiMedia®; Mumbai, India) with 4% iodine (Remel; Lenexa, Kansas, USA). The mixture was incubated at 42°C for 2 days. After approximately 24 h of incubation, the broth mixture was streaked onto Xylose Lysine Tergitol‐4 (XLT‐4) and Brilliant Green (BG) agars (Hardy Diagnostics; Santa Maria, California, USA). The XLT‐4 and BG agars were then incubated at 37°C for 24 h. The culture plates were observed for characteristic colonies of *Salmonella* spp. After 48 h of incubation, a second sample from the tetrathionate broth mixture was plated onto XLT‐4 and BG agars, incubated and observed for a characteristic growth of *Salmonella* spp. In addition to the targeted bacteria, any bacterial colonies observed were identified using a MALDI‐TOF Mass Spectrometer (Bruker; Billerica, Massachusetts, USA) following manufacturer's recommendations and recorded for data analysis.

### 2.4 Antimicrobial susceptibility

The antimicrobial resistance profile was determined on 32 *Escherichia coli* isolates recovered from the 36 sample pools, using the Kirby Bauer Disk Diffusion method (Hudzicki, 2009). A total of 13 different antimicrobial agents were tested: amikacin, amoxicillin‐clavulanate, ampicillin, azithromycin, cefoxitin, chloramphenicol, ciprofloxacin, gentamicin, kanamycin, meropenem, nalidixic acid, tetracycline and trimethoprim‐sulphamethoxazole. Antibiotic susceptibility was interpreted based on Clinical and Laboratory Standards Institute (CLSI) M100 guidelines for Enterobacterales (Weinstein et al., 2020).

### 2.5 Data analysis

The prevalence for the targeted microorganisms, *Campylobacter* spp., *Cryptosporidium* spp., *Giardia* spp., *Salmonella* spp. and antimicrobial resistant *E. coli*, and non‐targeted microorganisms was calculated according to Bush et al. (1997). The Sterne's exact method, using Quantitative Parasitology 3.0, was performed to calculate 95% confidence intervals (CIs) (Reiczigel, 2003; Rozsa et al., 2000).

## 3 RESULTS

### 3.1 Parasitic and bacterial examinations

Targeted pathogens, *Campylobacter* spp. (0/180; 0.0%; 95% CI: 0.0–2.1%), *Cryptosporidium* spp. (0/204; 0.0%; 95% CI: 0.0–1.8%), *Giardia* spp. (0/204; 0.0%; 95% CI: 0.0–1.8%) and *Salmonella* spp. (0/180; 0.0%; 95% CI: 0.0–2.1%), were not identified in the faecal samples examined (Tables 1, 2, 3, 4).

**TABLE 4:** Intestinal bacteria identified in pooled faecal samples of Canada geese inhabited in North‐Central Oklahoma

		Sampling locations (total number of pooled samples)										
		Stillwater, Payne county (*n* = 72)					Edmond and Oklahoma City, Oklahoma county (*n* = 78)				Broken Arrow, Tulsa county (*n* = 30)	
Bacteria	Family	L‐1 (*n* = 11)	L‐2 (*n* = 6)	L‐3 (*n* = 16)	L‐4 (*n* = 18)	L‐5 (*n* = 21)	L‐6 (*n* = 4)	L‐7 (*n* = 39)	L‐8 (*n* = 24)	L‐9 (*n* = 11)	L‐10 (*n* = 11)	L‐11 (*n* = 19)
*Aeromonas* spp.	Aeromonadaceae		X	X	X			X				
*Bacillus* spp.	Bacillaceae	X	X	X	X	X	X	X	X	X	X	X
*Exiguobacterium* sp.								X				
*Fictibacillus* sp.					X							
*Lysinibacillus* sp.											X	X
*Clostridium tertium*	Clostridiaceae	X					X				X	X
*Comamonas* sp.	Comamonadaceae							X				
*Cronobacter* sp.	Enterobacteriaceae		X	X		X		X			X	
*Escherichia coli*		X	X	X	X	X	X	X	X	X	X	X
*Enterobacter* spp.				X	X			X	X			X
*Escherichia* spp. *Non E. coli)*									X			X
*Klebsiella pneumoniae*								X				
*Klebsiella variicola*		X			X							
*Kosakonia cowanii*		X			X	X		X				X
*Leclercia adecarboxylata*								X				
*Pantoea* spp.				X	X	X						X
*Plesiomonas shigelloides*									X			
*Raoultella ornithinolytica*			X									
*Serratia marcescens*					X	X						
*Enterococcus* spp.	Enterococcaceae	X			X		X	X	X	X		X
*Curtobacterium* sp.	Microbacteriaceae				X							
*Arthrobacter gandavensis*	Micrococcaceae				X							
*Pseudomonas* spp.	Pseudomonadaceae				X	X						X
*Candida lusitaniae*	Saccharomycetaceae									X		
alpha *Streptococcus* spp.	Streptococcaceae							X				

*Note*: An ‘X’ demonstrates that the bacteria listed were cultured and identified from the corresponding location.

Faecal examinations, however, revealed various other parasites and bacteria (Tables 1, 2, 3, 4). Overall, the most commonly detected parasite stage was coccidian oocysts (153/204; 75.0%; 95% CI: 68.6–80.7%), followed by ascarid eggs (83/204; 40.7%; 95% CI: 34.2–47.5%), strongylid eggs (38/204; 18.6%; 95% CI: 13.9–24.6%), *Capillaria* spp. eggs (3/204; 1.5%; 95% CI: 0.3–4.4%), *Strongyloides* spp. eggs (3/204; 1.5%; 95% CI: 0.3–4.4%), cestode eggs (2/204; 1.0%; 95% CI: 0.04–3.7%) and schistosome eggs (1/204; 0.5%; 95% CI: 0.0–3.0%). Coccidia, ascarid and strongylid infections were observed in all three counties, whereas cestode and *Strongyloides* spp. infections were identified in Payne and Oklahoma counties. *Capillaria* spp. and schistosome infections were found only in Oklahoma county and Payne county, respectively. Of 153 faecal samples that contained coccidian oocysts, one sample obtained from the L‐7 in Oklahoma City, Oklahoma county demonstrated *Isospora* spp. oocysts (Table 2). A variety of bacterial species were also identified by bacterial cultures; *Bacillus* spp. and generic *E. coli* were recovered from all locations where faecal samples were collected (Table 4).

### 3.2 Antimicrobial susceptibility

Of the 32 *E. coli* isolates examined for antimicrobial resistance, 17 isolates (53.1%; 95% CI: 35.8–70.5%) demonstrated resistance to at least one antimicrobial agent. A total of 13 isolates (40.6%; 95% CI: 24.7–58.0%) were resistant to one antimicrobial agent; 12 isolates were resistant to azithromycin and one isolate was resistant to tetracycline. Three isolates (9.4%; 95% CI: 2.6–24.7%) were resistant to two antimicrobial agents belonging to different classes (azithromycin and tetracycline; azithromycin and kanamycin; azithromycin and ampicillin) and one isolate (3.1%; 95% CI: 0.17–16.6%) recovered from the L‐3 in Stillwater, Payne county was resistant to five antimicrobial agents, including azithromycin, amoxicillin‐clavulanate, ampicillin, chloramphenicol and tetracycline. Many *E. coli* isolates (16 of 32 isolates; 50.0%; 95% CI: 32.6–67.4%) showed resistance to azithromycin (Table 5).

**TABLE 5:** Antimicrobial susceptibilities of 32 *E. coli* isolates recovered from the faecal samples of Canada geese residing in 3 counties of North‐Central Oklahoma

	**Payne county (*n* = 11)**			**Oklahoma county (*n* = 15)**			**Tulsa county (*n* = 6)**			**Total (*n* = 32)**		
	Susceptible *n* (%) 5% CI	Intermediate *n* (%) 5% CI	Resistant *n* (%) 5% CI	Susceptible *n* (%) 95% CI	Intermediate *n* (%) 95% CI	Resistant *n* (%) 95% CI	Susceptible *n* (%) 95% CI	Intermediate *n* (%) 95% CI	Resistant *n* (%) 95% CI	Susceptible *n* (%) 95% CI	Intermediate *n* (%) 95% CI	Resistant *n* (%) 95% CI
Amikacin	11 (100.0) 73.6–100.0	0 (0.0) 0.0–26.5	0 (0.0) 0.0–26.5	15 (100.0) 77.8–100.0	0 (0.0) 0.0–22.2	0 (0.0) 0.0–22.2	5 (83.3) 41.1–99.1	1 (16.7) 0.86–58.9	0 (0.0) 0.0–41.1	31 (96.9) 83.4–99.8	1 (3.1) 0.17–16.6	0 (0.0) 0.0–10.5
Amoxicillin‐clavulanate	4 (36.4) 13.5–66.7	6 (54.6) 26.5–80.0	1 (9.1) 0.47–40.4	10 (66.7) 39.7–85.8	5 (33.3) 14.2–60.3	0 (0.0) 0.0–22.2	4 (66.7) 27.1–93.7	2 (33.3) 6.3–72.9	0 (0.0) 0.0–41.1	18 (56.3) 39.0–72.2	13 (40.6) 24.7–58.0	1 (3.1) 0.17–16.6
Ampicillin	8 (72.7) 40.5–92.1	1 (9.1) 0.47–40.4	2 (18.2) 33.4–50.0	10 (66.7) 39.7–85.8	5 (33.3) 14.2–60.3	0 (0.0) 0.0–22.2	6 (100.0) 58.9–100.0	0 (0.0) 0.0–41.1	0 (0.0) 0.0–41.1	24 (75.0) 57.7–87.8	6 (18.8) 8.5–35.8	2 (6.3) 1.1–20.0
Azithromycin	5 (45.5) 20.0–73.5	0 (0.0) 0.0–26.5	6 (54.6) 26.5–80.0	9 (60.0) 33.2–80.9	0 (0.0) 0.0–22.2	6 (40.0) 19.1–66.8	2 (33.3) 6.3–72.9	0 (0.0) 0.0–41.1	4 (66.7) 27.1–93.7	16 (50.0) 32.6–67.4	0 (0.0) 0.0–10.5	16 (50.0) 32.6–67.4
Cefoxitin	10 (90.9) 59.6–99.5	1 (9.1) 0.47–40.4	0 (0.0) 0.0–26.5	15 (100.0) 77.8–100.0	0 (0.0) 0.0–22.2	0 (0.0) 0.0–22.2	6 (100.0) 58.9–100.0	0 (0.0) 0.0–41.1	0 (0.0) 0.0–41.1	31 (96.9) 83.4–99.8	1 (3.1) 0.17–16.6	0 (0.0) 0.0–10.5
Chloramphenicol	9 (81.8) 50.0–96.7	1 (9.1) 0.47–40.4	1 (9.1) 0.47–40.4	15 (100.0) 77.8–100.0	0 (0.0) 0.0–22.2	0 (0.0) 0.0–22.2	6 (100.0) 58.9–100.0	0 (0.0) 0.0–41.1	0 (0.0) 0.0–41.1	30 (93.8) 80.0–98.9	1 (3.1) 0.17–16.6	1 (3.1) 0.17–16.6
Ciprofloxacin	11 (100.0) 73.6–100.0	0 (0.0) 0.0–26.5	0 (0.0) 0.0–26.5	15 (100.0) 77.8–100.0	0 (0.0) 0.0–22.2	0 (0.0) 0.0–22.2	5 (83.3) 41.1–99.1	1 (16.7) 0.86–58.9	0 (0.0) 0.0–41.1	31 (96.9) 83.4–99.8	1 (3.1) 0.17–16.6	0 (0.0) 0.0–10.5
Gentamicin	11 (100.0) 73.6–100.0	0 (0.0) 0.0–26.5	0 (0.0) 0.0–26.5	15 (100.0) 77.8–100.0	0 (0.0) 0.0–22.2	0 (0.0) 0.0–22.2	6 (100.0) 58.9–100.0	0 (0.0) 0.0–41.1	0 (0.0) 0.0–41.1	32 (100.0) 89.5–100.0	0 (0.0) 0.0–10.5	0 (0.0) 0.0–10.5
Kanamycin	2 (18.2) 33.4–50.0	9 (81.8) 50.0–96.7	0 (0.0) 0.0–26.5	3 (20.0) 5.7–46.6	12 (80.0) 53.4–94.3	0 (0.0) 0.0–22.2	0 (0.0) 0.0–41.1	5 (83.3) 41.1–99.1	1 (16.7) 0.86–58.9	5 (15.6) 6.4–32.6	26 (81.3) 64.2–91.5	1 (3.1) 0.17–16.6
Meropenem	11 (100.0) 73.6–100.0	0 (0.0) 0.0–26.5	0 (0.0) 0.0–26.5	15 (100.0) 77.8–100.0	0 (0.0) 0.0–22.2	0 (0.0) 0.0–22.2	6 (100.0) 58.9–100.0	0 (0.0) 0.0–41.1	0 (0.0) 0.0–41.1	32 (100.0) 89.5–100.0	0 (0.0) 0.0–10.5	0 (0.0) 0.0–10.5
Nalidixic acid	10 (90.9) 59.6–99.5	1 (9.1) 0.47–40.4	0 (0.0) 0.0–26.5	14 (93.3) 69.8–99.6	1 (6.7) 0.35–30.2	0 (0.0) 0.0–22.2	5 (83.3) 41.1–99.1	1 (16.7) 0.86–58.9	0 (0.0) 0.0–41.1	29 (90.6) 75.3–97.4	3 (9.4) 2.6–24.7	0 (0.0) 0.0–10.5
Tetracycline	10 (90.9) 59.6–99.5	0 (0.0) 0.0–26.5	1 (9.1) 0.47–40.4	12 (80.0) 53.4–94.3	1 (6.7) 0.35–30.2	2 (13.3) 2.4–39.7	6 (100.0) 58.9–100.0	0 (0.0) 0.0–41.1	0 (0.0) 0.0–41.1	28 (87.5) 71.9–95.6	1 (3.1) 0.17–16.6	3 (9.4) 2.6–24.7
Trimethoprim‐sulphamethoxazole	11 (100.0) 73.6–100.0	0 (0.0) 0.0–26.5	0 (0.0) 0.0–26.5	15 (100.0) 77.8–100.0	0 (0.0) 0.0–22.2	0 (0.0) 0.0–22.2	6 (100.0) 58.9–100.0	0 (0.0) 0.0–41.1	0 (0.0) 0.0–41.1	32 (100.0) 89.5–100.0	0 (0.0) 0.0–10.5	0 (0.0) 0.0–10.5

## 4 DISCUSSION

To the best of our knowledge, this is the first study investigating the prevalence of zoonotic pathogens, *Cryptosporidium* spp.*, Campylobacter* spp.*, Giardia* spp. and *Salmonella* spp. and antimicrobial resistant *E. coli* in the faeces of Canada geese residing in North‐Central Oklahoma, US. While the current study did not detect any targeted zoonotic pathogens in the faecal samples examined, previous studies in Canada geese revealed the prevalence as high as 51.8% for *Campylobacter* sp. (Fallacara et al., 2001), 90.0% for *Cryptosporidium* spp. (Kassa et al., 2004), 100.0% for *Giardia* spp. (Graczyk et al., 1998) and 2.3% for *Salmonella* spp. (Fallacara et al., 2004). The presence of pathogens in Canada geese can vary due to many factors including changes in migratory patterns (Kwon et al., 2017) and seasonal differences (Vogt et al., 2018). Traditionally, Canada geese breed during summer months in Canada and migrate and stay over winter in the United States; however, an increasing abundance of high‐quality habitat and a lack of predators for geese in the United States has resulted in the growth of resident (non‐migratory) geese populations (Environment Canada, 2011; USFWS, 2005; Zhou et al., 2004). In contrast to migratory Canada geese, resident Canada geese may have a decreased risk of being exposed to environments contaminated with zoonotic pathogens since they only fly locally for food sources (McPhaul, 2016). Similar to our findings, a previous study on 234 faecal samples collected from resident Canada geese in North Carolina did not detect any evidence of *Giardia* spp. infection (Ayers et al., 2014). Although previous surveys in Canada geese did not directly compare the prevalence of pathogens between migratory and non‐migratory geese, different migratory patterns may possibly influence the prevalence.

Culture of the pooled faecal samples in the current study yielded multiple bacterial species, majority of which belonged to either Bacillaceae or Enterobacteriaceae families (Table 4). The bacterial species detected were most likely commensals of the intestinal tract as similar species have been reported in faecal samples of different animal species (Cinquepalmi et al., 2012; Zhao et al., 2020). Many of these bacteria can cause opportunistic infections. Some of the bacterial species such as *Enterococcus* spp., which were detected from seven of the 11 locations studied, are significant in that they are inherently resistant to different antibiotics and capable of causing human infections (Agudelo Higuita et al., 2014). Our study did not demonstrate the presence of targeted pathogenic bacteria such as *Salmonella* spp. or *Campylobacter* spp. A low prevalence of *Salmonella* spp. in Canada geese faeces conforms with results of other studies conducted in North America (Converse et al., 1999; Fallacara et al., 2004; Vogt et al., 2018). This could be due to a low interaction of bird populations with contaminated environments, such as infected livestock, and a generally higher sanitary condition of locations sampled in the current study (dos Santos et al., 2020; Hernandez et al., 2003). Similarly, the prevalence of *Campylobacter* spp. in wild birds has been highly variable depending on geographic regions (Kaakoush et al., 2015) and between indigenous birds compared to migratory birds (Kwon et al., 2017). It has also been reported that the prevalence of *Campylobacter* spp. can be influenced by the feeding characteristics of birds, with very low levels found in ground foraging granivores and arboreal/aerial insectivores (Waldenström et al., 2002). Additionally, in this study, for *Campylobacter* spp. culture, faecal samples were directly plated on selective media without bacterial enrichment. The failure to detect *Campylobacter* spp. could also be a limitation of this less sensitive direct culture approach compared to the enrichment culture protocol followed in other studies (Vogt et al., 2018). In addition to bacteria, yeast colonies (*Candida* spp.) were detected in one of the faecal sample pools from location L‐11. The detection of yeast in low frequency from faeces of Canada geese as well as other avian species has been reported (Buck, 1990; Glushakova et al., 2020). Yeasts, such as *Candida* spp., are commensal organisms but can cause opportunistic infections and pose potential risks to immune‐compromised humans (Blinkhorn et al., 1989; Rahmati et al., 2020; Wawrysiuk et al., 2018).

One of the interesting findings in our study with potential zoonotic significance was the identification of schistosome eggs in a faecal sample collected in Stillwater, Payne county. Schistosomes are parasitic flatworms that can cause cercarial dermatitis, also commonly known as ‘swimmer's itch’ in humans (Horák et al., 2015). The most frequently identified schistosome in Canada geese is *Anserobilharzia brantae*, which was formerly classified as *Trichobilharzia brantae* (Brant et al., 2013; Horák et al., 2015). There is considerable diversity among schistosomes that may initiate cercarial dermatitis; four schistosome genera with about 30 described species from mammals and 10 genera with about 67 species from avian have been reported. Of the diversified family of Schistosomatidae, species of *Trichobilharzia* appear to be the primary etiological agents for dermatitis outbreaks globally (Horák et al., 2015). However, an outbreak of cercarial dermatitis caused by *A. brantae* (called as *T. brantae* at that time) transmitted by Canada geese and the freshwater snail, *Gyraulus parvus*, has been reported in Colorado Springs, Colorado (Brant & Loker, 2009). *Anserobilharzia brantae* adults are found in the blood vessels of Canada geese and produce eggs that are passed into the faeces (Acha & Szyfres, 2003; Brant et al., 2013; Center for Disease Control and Prevention, 2019). When the egg reaches water, a ciliated miracidum is released and swims in search of a suitable molluscan intermediate host, *Gyraulus* freshwater snails (Acha & Szyfres, 2003; Brant et al., 2013; Center for Disease Control and Prevention, 2019; Horák et al., 2015). After the miracidia penetrate snail bodies and develop into a cercarial stage in several weeks, fully matured cercaria leave the snail and swim around while searching for a definitive host. Canada geese become infected while in water through skin penetration of cercariae (Acha & Szyfres, 2003; Center for Disease Control and Prevention, 2019). Humans accidentally contract cercariae cutaneously while swimming, wading and playing in contaminated water; dermatitis results from an immune reaction to the cercariae. To reduce the risk of developing cercarial dermatitis, avoiding swimming or wading in water where snails and birds are commonly observed is recommended (Acha & Szyfres, 2003; Center for Disease Control and Prevention, 2020). Unfortunately, confirmatory tests to identify genus and species of the observed schistosome were not conducted in the current study due to a lack of faecal sample after parasitic and bacterial examinations were performed.

Of note, coccidia was the most commonly detected gastrointestinal parasite in the faeces of Canada geese in this study. Although there is scant information about the clinical significance of coccidiosis in geese, the infection has been reported and appears to be a common finding (Fallacara et al., 2001; Greiner et al., 1981; Skene et al., 1981; Soulsby, 1982; Tuggle & Crites, 1984). Three different genera of intestinal coccidia, *Eimeria*, *Isospora* and *Tyzzeria*, have been reported in Canada geese (Skene et al., 1981). In order to differentiate the three coccidian genera morphologically, oocysts need to be cultured with 2.0–2.5% potassium dichromate solution to facilitate sporulation (Berto et al., 2007; Greiner et al., 1981). Although one sample obtained in Oklahoma City, Oklahoma county contained coccidian oocysts with two sporocysts, a morphological characteristic of *Isospora* spp. (Table 2) (Skene et al., 1981; Zajac & Conboy, 2012), further identification of coccidia was not performed in this study. None of these coccidian parasites in Canada geese are considered as zoonotic agents.

*Escherichia coli* isolates were generally susceptible to most antimicrobials tested. No consistent multidrug resistance patterns were observed among isolates collected from the different counties. There was a single *E. coli* isolate from Stillwater, Payne County that showed resistance to multiple antimicrobials including potentiated beta‐lactam, macrolide, phenicol and tetracycline classes, highlighting possible risks that these birds can pose as a source of potential harmful microbes. Most of the *E. coli* isolates showed resistance to azithromycin. Azithromycin is a macrolide antibiotic that has been suggested as a treatment for certain kinds of *E. coli* infections (Nitschke et al., 2012). In the current study, most of the resistant isolates (13/32) had a zone diameter of 12 mm which is the higher end of the range to be categorised as ‘resistant’. Zone diameters above 12 mm are considered ‘susceptible’. Further diagnostic work should be conducted to determine the genetic basis of the resistance to azithromycin.

There are some limitations in this study. Faecal samples were pooled for *Giardia* spp. and bacterial examinations due to financial and time constraints although it was not an ideal procedure to investigate the prevalence of microorganisms in geese as pool testing could provide only the prevalence of a flock or sampling location, instead of individual geese. In the current study, identification or tracking of individual Canada geese was not conducted. At every sampling visit, a single collection of faeces per goose was encouraged; however, multiple collections from the same goose could have occurred as Canada geese were capable of traveling from a location to another freely. Since faecal collection was conducted only during summer months (May through July) in 2019, our data in the current study did not consider seasonal or yearly differences. Additionally, one time faecal examination may not be sufficient to determine the prevalence of microorganisms in Canada geese due to intermittent shedding of *Giardia* spp. cysts (Zajac & Conboy, 2012), non‐patent infections and size of the faecal sample. Although specialised growth enrichment media are preferred for detecting fragile bacterial species such as *Campylobacter* spp., a direct culture approach was used in this study, which could have resulted in the failure to isolate these potential pathogens. Lastly, since only aerobic and microaerophilic culture protocols were used, detection of obligate anaerobic bacteria was not achieved. Metagenome or targeted genome sequencing‐based approaches will help in getting a more comprehensive understanding of the different pathogenic bacterial species.

In conclusion, the current study suggests that resident Canada geese observed in North‐Central Oklahoma may not play a major role in the transmission of zoonotic pathogens, such as *Campylobacter* spp., *Cryptosporidium* spp., *Giardia* spp. and *Salmonella* spp. The findings of multiple‐antimicrobial resistant *E. coli* and schistosome infections are potentially a public health concern although the prevalence was low in this study. As this study was relatively a small‐scale and seasonal study, additional larger scale studies over a longer period of time are recommended in the interest of keeping the public residential and recreational areas safe and monitoring the prevalence of zoonotic and antimicrobial resistant agents.

## AUTHOR CONTRIBUTIONS

YN was a major contributor in writing the manuscript, developed experimental protocols, analysed data and supervised faecal examinations for parasitic detection. MAL collected faecal samples, performed faecal examinations for parasitic and bacterial detections and analysed data. AR developed experimental protocols, analysed data and supervised faecal examinations for bacterial detection. NRK and ARL collected faecal samples and supported for faecal examinations. All authors read and approved the final manuscript.

## CONFLICT OF INTEREST

Authors declare no conflict of interest.

## ETHICS APPROVAL STATEMENT

The study did not require a regulatory approval by the Institutional Animal Care and Use Committee (IACUC) because animals were not directly or indirectly handled. All faecal samples were collected from the ground in the public areas.

### PEER REVIEW

The peer review history for this article is available at https://publons.com/publon/10.1002/vms3.791

## Data Availability

The data that support the findings of this study are available from the corresponding author, YN, upon reasonable request.
